# E‐SSSential questions and 3‐D mnemonic to diagnose dermatologic diseases

**DOI:** 10.1111/ddg.70258

**Published:** 2026-03-07

**Authors:** Falk Ochsendorf

**Affiliations:** ^1^ Goethe University Frankfurt, University Hospital, Clinic for Dermatology Venereology and Allergy Frankfurt am Main Germany

Dear editors,

I read Salava's correspondence with great interest.^1^ It was gratifying to find that the approach I have been practising for more than three decades and teaching to both students and physicians in further training,^2^ has also proven helpful to him. The proposed mnemonic ‘3 C’ corresponds to my own approach, which I refer to in German as the ‘3 S’ questions (‘Seit wann (= Since when  =  Course)? Symptome (= symptoms  =  complaints)? Salben (= salves/ointments as synonym for previous therapy  =  Cure). Everyone can decide for themselves which mnemonic aid (3C or 3S) they prefer. As stated in the correspondence this approach has several advantages, such as a clear structure, aid memoire of critical aspects of history taking and easy‐to‐learn basis for students.^1^

However, I would like to emphasise the clinical relevance of these questions (Table 1) and to comment on the more general aspects of the advantages of this approach.

**TABLE 1:** 3 C/3 S mnemonic approach and the clinical relevance of the respective questions.

3 C	3 S	Can help to classify as
**Course**	**Since when?**	Acute / chronic / relapsing disease, genodermatosis etc.
**Complains**	**Symptoms?**	Allergic / infectious / neoplastic etc.
**Cure**	**Salves?**	Primary skin changes / appearance changed by prior therapy / hint to a certain pathophysiology depending on the treatment response

1. Why exactly should these three questions be asked?

Because the answers to these questions cannot be deduced from the clinical examination – they cannot be seen! For example, scratch marks may indicate itching, but they can also exist independently of the skin disease and do not necessarily reflect its severity. Chills/fever cannot always be immediately recognized clinically. Everything else can be detected by inspecting the skin (see 3 D below;^2^)!

2. Why should only these three questions be asked initially?

Because this protects against errors! Unlike other disciplines, especially internal medicine, the initial anamnesis in dermatology should be brief (‘look first, talk later’).^2^ This prevents hypotheses or explanations put forward by the patient or previous practitioners from unintentionally leading to diagnostic bias.^3^ It can be difficult to break away from this.

3. What are the advantages of the 3S/3C approach?

The procedure is time‐effective! It enables you to make direct contact with the patient quickly, after which the examination begins. If, for example, the findings suggest an occupational contact dermatitis, taking a detailed medical history is helpful, but not necessary if the clinical findings indicate shingles. Therefore, the more comprehensive, targeted medical history is only taken after the findings have been recorded (see 3 D below).

In addition to the structured anamnestic procedure (3 S/3 C mnemonic), I would like to suggest the following 3 D mnemonic for diagnosing dermatological diseases, which has also proven useful in practice,^2^ (Table 2). After asking the 3S/3C questions, the following steps should be followed:

1. **Describe**. The description of the findings should be done systematic and structured (‘from the big picture to the small details’: location, distribution, shape, efflorescence) and a generally accepted nomenclature should be used (see,^4^ for English and,^2^ for German). Terms such as ‘skin changes’ or ‘skin lesions’ are unfortunately not helpful here and should be avoided!
2. **Differentiate** (i e. formulate differential diagnoses (DDs)/set up hypotheses)

**TABLE 2:** 3 D mnemonic for the diagnosis of dermatological diseases.^*^

Steps	Function and advantages
**Describe**	Forces to really look at the skin in detail: from the big picture to the small details: location, distribution, shape, efflorescence: Induces cognitive activities: what happens in the skin?
**Differentiate**	Formulate DDs/hypothesis: which diseases may have this course, these symptoms and these findings? Activates Type I reasoning*
**Diagnose/deliberate reflection**	Rule out hypotheses, which do not fit for one reason or another to the suspected DD (history, clinical finding); identify diagnostic measures to rule out/confirm the remaining hypotheses/DDs by history and/or laboratory/technical/histological or other investigations. Activates Type II reasoning to combat bias*

* Type I describes intuitive and implicit mental reasoning which is fast and has a lower cognitive load. On the contrary, type II reasoning illustrates analytic thinking with carefully stepwise evaluation and deduction; for details see.^2^, ^8^

Based on the information in the 3‐S/3‐C medical history and the clinical findings, differential diagnoses/hypotheses are formulated: what must have happened in the skin to result in the observed finding to arise in the described context? Here it is helpful here to think ‘broadly’, i. e. to formulate as many DDs/hypotheses as possible.

3. **Diagnose**/deliberate reflection (diagnostic measures to confirm or exclude the DDs)

To avoid cognitive bias, it is advisable to look for arguments *against* the initial visual diagnosis. This allows either a definitive diagnosis to be made or only a few possible differential diagnoses to remain.^5^ This process, deliberate reflection, has been shown to improve clinical reasoning and diagnostic accuracy in general,^6^, ^7^ and in dermatology.^5^

The remaining suspected diagnoses are now confirmed or ruled out using appropriate measures, such as taking a medical history, carrying out technical examinations or performing biopsies. If the diagnosis remains unclear after receiving the results of the respective tests it is advisable to repeat these three steps, starting with a new assessment of the findings. Based on my experience, the second step, searching for differential diagnoses, is often skipped. This is often followed by broad, mostly untargeted diagnostics. This often leads to pathological findings that are irrelevant to the specific case but which require further clarification and can sometimes have unfavorable consequences for the patients. Consistent adherence to these three steps protects against this.

In summary, the practical relevance of the 3 C or 3 S approach should be emphasised, along with a theoretical justification. To make successful dermatologic diagnoses it is recommended that the 3 C/3 S questions are followed by a 3‐D approach in daily practice.

## CONFLICTS OF INTEREST STATEMENT

None
